# Assessment of weight and height of patients with primary immunodeficiency disorders and group of children with recurrent respiratory tract infections

**DOI:** 10.1186/s12865-020-00372-x

**Published:** 2020-07-16

**Authors:** Karolina Pieniawska-Śmiech, Kamil Bar, Mateusz Babicki, Karol Śmiech, Aleksandra Lewandowicz-Uszyńska

**Affiliations:** 1 Student Research Circle at 3rd Department and Clinic of Paediatrics, Immunology and Rheumatology of Developmental Age, Wroclaw Medical University, Wrocław, Poland; 2 Department of Clinical Immunology, Wroclaw Medical University, Wrocław, Poland; 3 J.Gromkowski Regional Specialist Hospital, Wrocław, Poland; 4 1st Department and Clinic of Paediatrics, Allergology and Cardiology, Wroclaw Medical University, Wrocław, Poland; 5 Jan Mikulicz-Radecki University Teaching Hospital, Wrocław, Poland; 6 Department of Family Medicine, Wroclaw Medical University, Wrocław, Poland; 7 Department of Cardiology, Regional Specialist Hospital, Research and Development Center, Wrocław, Poland; 8 3rd Department and Clinic of Paediatrics, Immunology and Rheumatology of Developmental Age, Wroclaw Medical University, Wrocław, Poland

**Keywords:** Primary immunodeficiency, Recurrent respiratory tract infections, Physical growth, Growth assessment

## Abstract

**Background:**

Primary immunodeficiences (PIDs) are a group of chronic, serious disorders in which the immune response is insufficient. In consequence, it leads to an increased susceptibility to infections. Up to date, there are about 350 different disorders classified in that group. There are also patients suffering from recurrent respiratory tract infections (RRTI), however that group doesn’t present any abnormalities in terms of conducted immunological tests. Many factors, including medical, can have an impact on physical development of a child. Data such as birth weight and length, also weight, height, BMI during admission to the hospital were collected from 195 patients’ medical histories from their hospitalization at Clinical Immunology and Paediatrics Ward of J. Gromkowski Hospital in Wrocław. Investigated groups included patients with PIDs, RRTI and a control group of healthy children. Our purpose was to evaluate the physical growth of children with PID and children with RRTI by assessment of their height and weight. All of parameters were evaluated using centile charts, suitable best for the Polish population.

**Results:**

The lowest mean birth weight and height was found among the PIDs patients group. Children with PIDs during hospitalization had statistically relevant lower mean weight than the control group and almost 18% of them had their height situated below 3rd percentile. The statistically relevant differences have been found between them and RRTI group in terms of weight, height and nutritional status. The statistically significant difference was detected between the nutritional status of PID and control group.

**Conclusions:**

There is a higher percentage of PID patients with physical growth abnormalities in comparison to healthy children. Our findings indicate a need for further investigation of immune system irregularities and their influence on physical growth of children.

## Background

Primary immunodeficiencies (PIDs) are uncommon, chronic and severe disorders of the immune system in which patients cannot mount a sufficiently protective immune response, leading to an increased susceptibility to infections, as well as autoimmune or neoplastic diseases [1]. About 350 nosological entities were characterized and classified as PIDs up to now [2].

More than six serious diseases a year are defined as recurrent respiratory tract infections (RRI) [3]. During the development of infections many different immunologic disturbances can occur, hence they are a difficult diagnostic challenge. Among the predisposing factors immune system deficiencies should be considered [4]. Early and accurate diagnosis is essential to ensure that the appropriate treatment is given and to minimise irreversible changes [5].

Although care standards of children with PIDs were elaborated, still physical abnormalities in their physical development, such as depressed weight or height gain, can be observed, which according to The Jeffrey Modell Foundation, are also among the warning signs of PIDs [6, 7]. We decided to assess weight, height and nutritional status of children with PID, as well as children with recurrent respiratory tract infections, and compare these important clinical features with data from healthy children. We wanted to draw attention to the need of regular assessment of these parameters and evaluation of physical growth, particularly in group of children with PID. We tried to assay if there are differences between anthropometric parameters of children with PID and children with RRTI. Collaterally, we attempted to discover if there were abnormalities in birth weight and length in group of patients with PID.

While many studies have investigated higher frequency of malnutrition and anthropometric values abnormalities in some of diseases from the PID spectrum, we have not come across a paper that deals with assessment of nutritional status of patients with PID beside patients with recurrent respiratory tract infections and healthy children.

## Results

### Birth weight and length

**Fig. 1:**
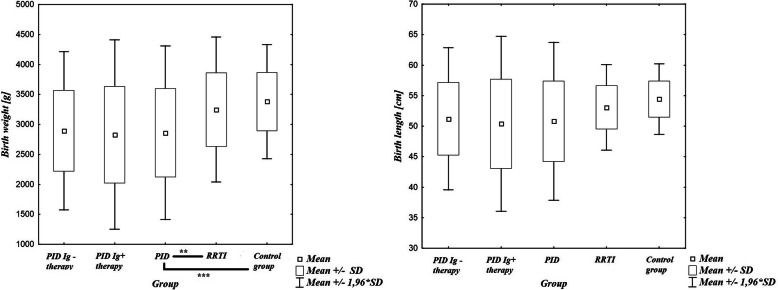
Birth weight and length analysis results as mean values ± standard deviations, PID divided into group with immunoglobulin substitution (PID Ig+therapy) and without (PID Ig-therapy). *-*p* < 0.05; ** -*p* <0.01; ***-*p* < 0.001

Birth weight values of 185 patients were available for analysis. The highest standard deviation (SD = 738) and the lowest mean birth weight (mean 2860.1 g) referred to patients with PID (Fig. 1). In addition, patients treated with immunoglobulin (PID Ig + therapy) were born with a little less mean birth weight (mean 2829.07 g) and higher SD (SD = 805.47) than PID patients without therapy (PID Ig -therapy; mean 2893.6; SD = 672.25), but difference between these groups was not statistically relevant (*p* = 0,75). 26.92% (*n* = 14) of children with PID were born with weight under 2500 g, while in the RRTI group only 5.08% (*n* = 3, among 59 available data) and in the control group 2.86% (*n* = 2, among 70 available data) of children had the same low weight score. There were no statistically significant differences between males and females in the PID group (*p* = 0,38). There was a significant statistical difference between PID group and RRTI group (p = 0,003), as well as between PID and control group (p = 0,000007). Such difference was not observed between RRTI group and control group (p = 0,167). 10 of 14 children, who where born with weight under 2500 g, were diagnosed with predominantly antibody deficiencies.

Birth length values were collected from 156 patients. Children with PID had the lowest mean birth length (mean = 50.8 cm; median = 52 cm) and the highest standard deviation among all groups (SD = 6.69); when dividing into groups with or without substitution of immunoglobulin, the highest SD (7.31) and the lowest mean birth length (h = 50.4 cm) related to PID Ig + therapy (Fig. 1).

### Weight during hospitalization

**Fig. 2:**
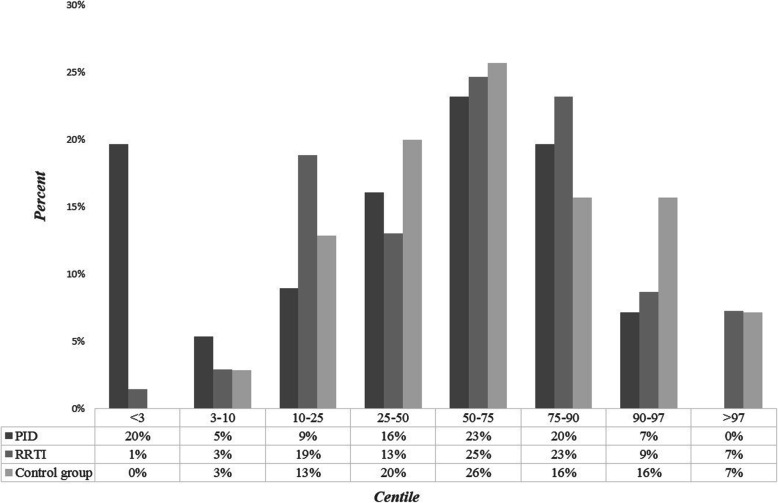
Centiles percent distribution of certain patients groups actual weights, according to Polish standards. A significant statistical difference was found between weight centiles of children with PID and control group (*p* = 0,003) and between PID and RRTI group (*p* = 0,013)

Weight during hospitalization data (patients were hospitalized at different ages) were available for all the patients (*n* = 195). They were assigned to certain centiles, based on the Polish growth standards (Fig. 2). Almost 20% of patients with PID (*n* = 11) had weight under 3rd percentile, 1,5% (n = 1) of the RRTI group. There was a statistically significant difference in weight centiles between these 2 groups (Chi^2 Pearson test, *p* = 0,013). Relevantly more often weight under 3rd percentile affected children with PID Ig + therapy (*n* = 10) than those without substitution (PID Ig-therapy, n = 1), although there were no statistically significant differences between those groups (Pearson Chi^2 test *p* = 0,158). A significant statistical difference was also found between weight centiles of children with PID and control group (p = 0,003). Such difference was not found between RRTI and control group (p = 0,63). There were no statistically significant differences between males and females, both in the PID group (p = 0,689) and the RRTI group (p = 0,798). In case of PID group, weight under 3rd percentile most often affected patients between 6 and 12 years old.

### Height during hospitalization

**Fig. 3:**
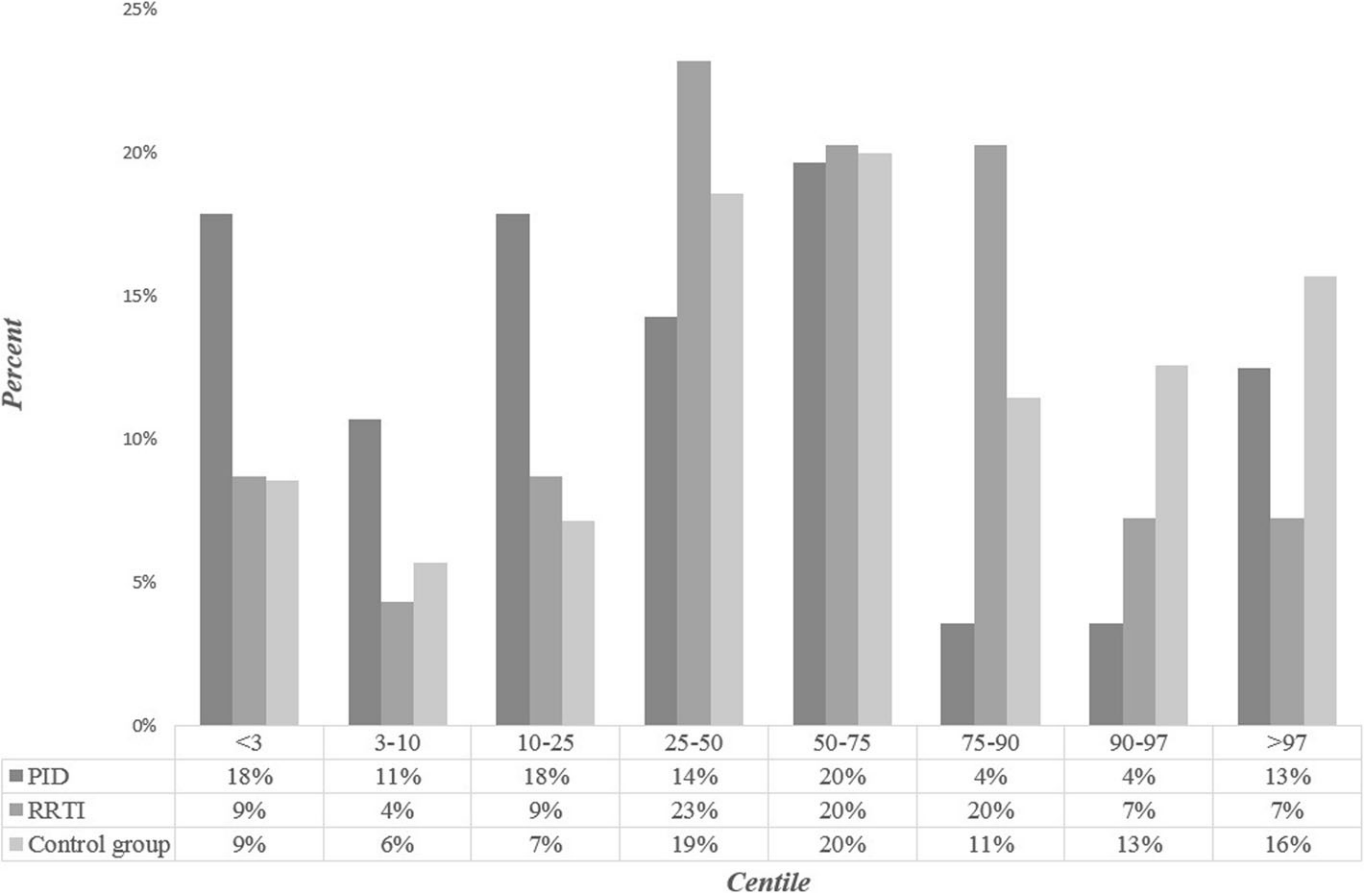
Centiles percent distribution of certain patients groups height, according to Polish standards. There was a statistically significant difference between height assigned to certain centiles of PID and RRTI group (*p* = 0,031)

**Fig. 4:**
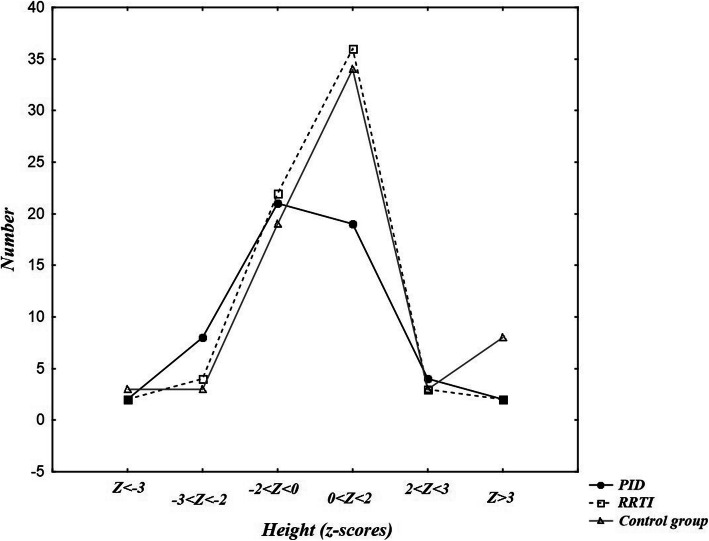
Z-score values of height of the PID, RRTI and control group patients. There were no statistically significant differences between PID and RRTI group (*p* = 0,362) or PID and control group (*p* = 0,1) in terms of height Z-score

Height during hospitalization data were available as well for all the patients (*n* = 195). They were classified to certain centiles according to both Polish (Fig. 3) and WHO standards. According to Polish standards 18% PID patients (*n* = 10) and 9% RRTI patients (*n* = 6) were classified in the < 3 centile compartment. In the 3–10 percentile interval there were 6 patients in the PID group (11%) and 3 in the RRTI group (4%). There was a statistical difference between height assigned to certain centiles of PID and RRTI group (*p* = 0,03) with the Polish standards. According to WHO’s, 10 patients (17,86%) from the PID group were situated below 3rd percentile of height, same with the z-score < −2SD (Fig. 4). Z-score < −2SD more frequently referred to the Ig + therapy PID patients (*n* = 7) than to patients without substitution (*n* = 3), although it wasn’t statistically relevant (*p* = 0,502). There was a statistically relevant difference detected between female and male height Z-score from the PID group (p = 0,020), but no in the RRTI group (p = 0,62). In the PID group, there were 5 girls with height between -2SD and -3SD z-score and 0 girls with height under -3SD z-score, while 3 boys with PID had height between -2SD and -3SD z-score and 2 had height under -3SD z-score. 6 patients (8.7%) from the RRTI group had their height below Z-score < −2SD. There were no statistically significant differences between PID and RRTI group in terms of height Z-score (*p* = 0,362) or between PID and the control group both in the height centiles of Polish standards (p = 0,096) and Z-score (p = 0,1).

### BMI

**Fig. 5:**
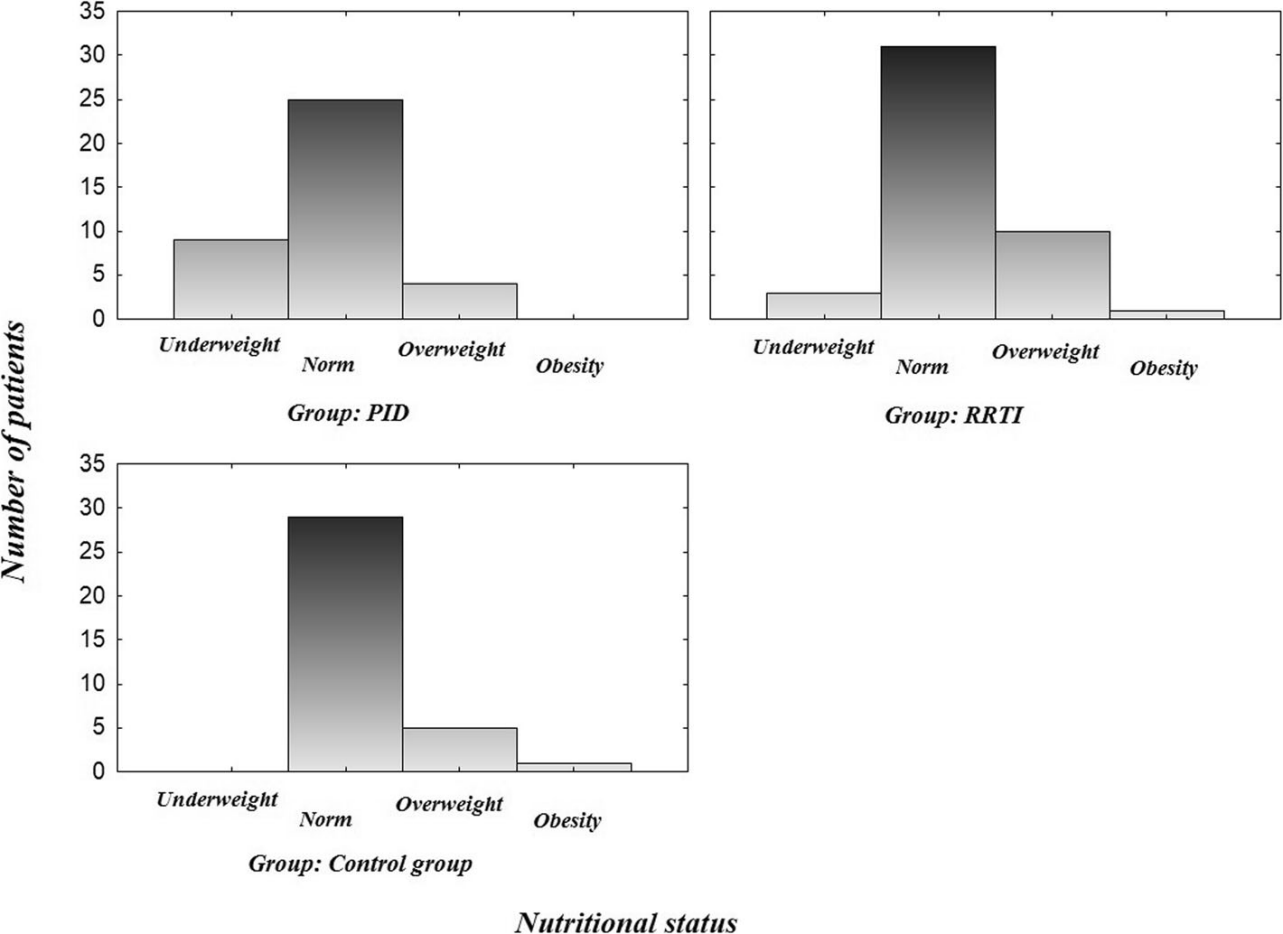
Nutritional status of participants aged 3-18 years old divided into groups. The statistically significant differences were detected between the nutritional status of PID patients and RRTI group (*p* = 0,023), as well as PID and control group (*p* = 0,016)

Based on data collected from all of the patients the BMIs were calculated and classified to certain nutritional status of Polish centile charts standards (Fig. 5). Nutritional status was possible to define using centile charts for age 3–18 years old (PID *n* = 38, RRTI *n* = 45, control group *n* = 35). It was within normal for most of the patients (PID-65.79%, *n* = 25, RRTI-68.89%, *n* = 31, control group-82.86%, *n* = 29). 9 patients over 3 years old from the PID group were underweight (23.68%; 16.07% of all participants). In the RRTI group 3 patients over 3 years old were underweight (6.67, 4.35% of all participants). 4 PID patients and 10 RRTI were diagnosed with overweight. Obesity was observed in one person in the RRTI group, whereas in the PID group no sign of obesity was found. The difference between these 2 groups was statistically significant (*p* = 0,023). The statistically significant difference was also observed between the nutritional status of PID patients and the control group (p = 0,016), but was not detected between RRTI and control group (p = 0,316) (Fig. 5). There was no statistically relevant difference between genders in the PID group (p = 0,058) and in the RRTI group (p = 0,194). In the PID group, underweight was more common in girls (*n* = 8) than boys (*n* = 1).

**Fig. 6:**
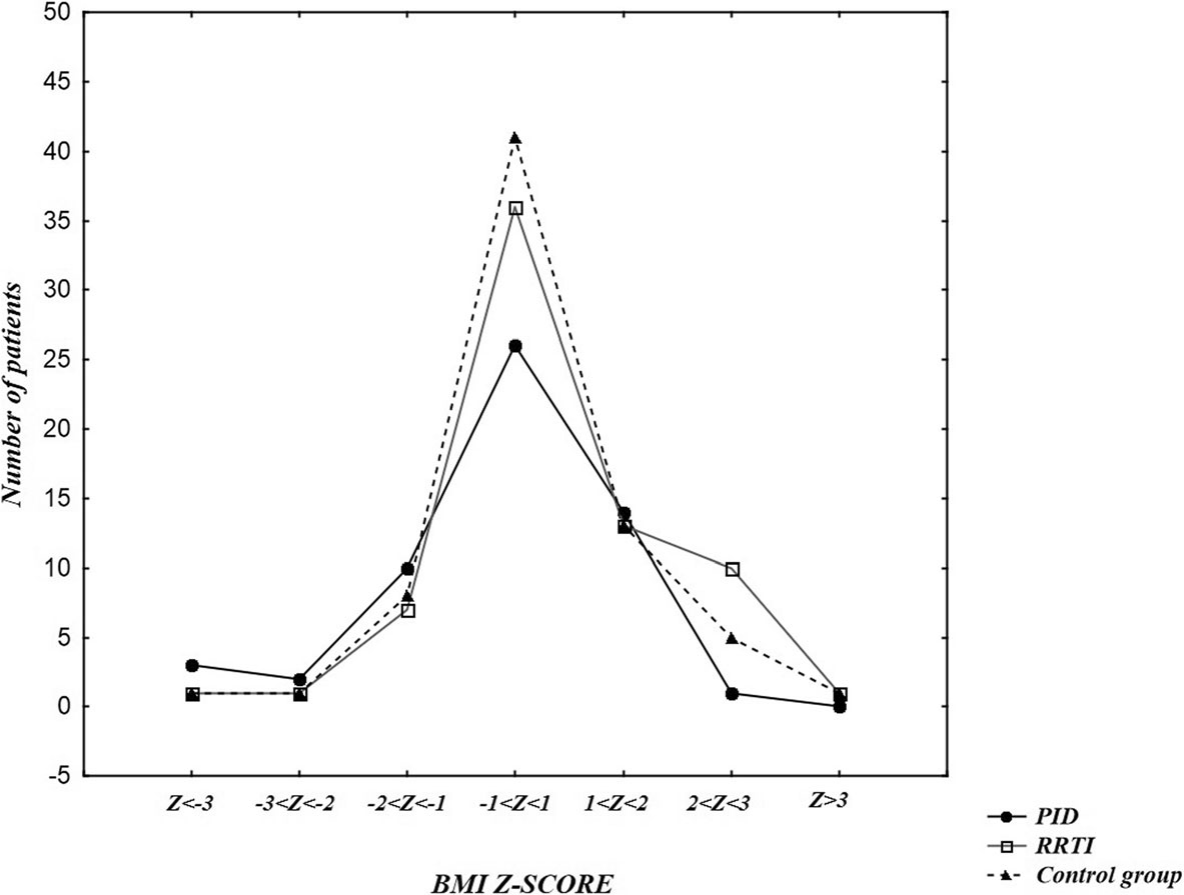
BMIs Z-score of the PID, RRTI and control group. There was no statistically relevant difference in z-score BMI between PID and control group (*p* = 0,307), as well as between PID and RRTI (*p* = 0,1)

BMI was also assessed using the z-score based on WHO standards for all participants (Fig. 6). Z-score < − 2 was detected among 5 people in PID group (8.2%), 3 of them had BMI < -3 z-score. Among these 5 people, 2 remained without immunoglobulin substitution by the time of study, the other 3 had such treatment. Three from that group were under 3 years old, so evaluation of nutritional status with Polish standards wasn’t possible. One patient was diagnosed with ataxia-telangiectasia (z-score < − 3), one with Nijmegen breakage syndrome (− 3 < z-score < − 2), three had predominantly antibody deficiencies (*n* = 2 z-score < − 3; *n* = 1–3 < z-score < − 2) with no other already diagnosed conditions. Z-score between − 1 and -2SD included 10 patients with PID (17.86%). 14 patients (25%) had a z-score between + 1 and + 2SD and only one patient (1.64%) had BMI >2SD.

In RRTI group 2 patients (2.9%) had Z-score < −2SD, 7 patients (10.14%) had Z-score between -2SD and -1SD, 13 people (18.84%) 1SD < Z-score < 2SD and 11 patients (15.94%) > 2SD. There was no statistically relevant difference in z-score BMI between PID and control group (*p* = 0,307), as well as between PID and RRTI (*p* = 0,1).

**Fig. 7:**
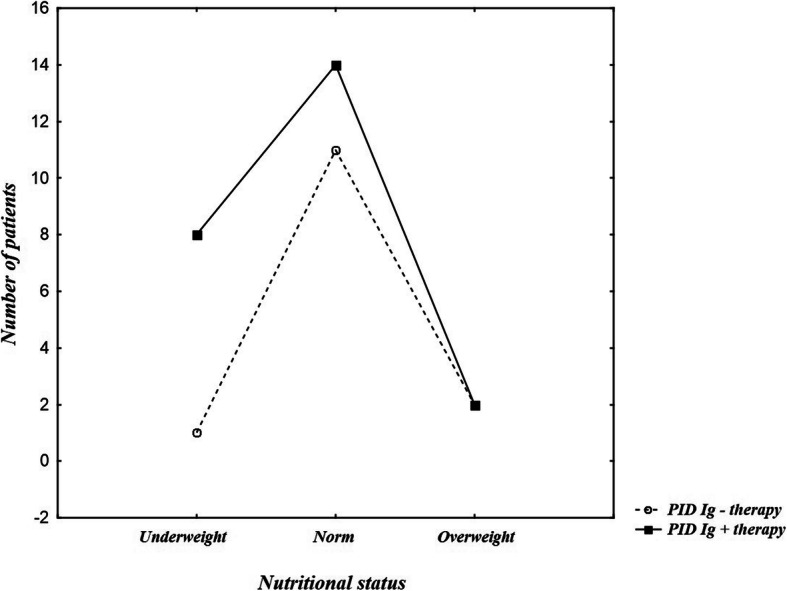
Nutritional status of PID patients group divided into Ig+therapy and Ig-therapy groups

There was no statistically relevant difference in nutritional status using Polish standards between PID patients with or without immunoglobulin substitution (p = 0,143), although it’s worth noting that underweight more frequently referred to Ig + therapy patients (*n* = 8; 33.33%), than to Ig-therapy (*n* = 1; 7.14%) (Fig. 7). No statistically relevant difference was found either in case of BMI Z-score (p = 0,163).

Among 9 underweight patients from PID group (ages 3–18), 4 people had been diagnosed with ataxia-telangiectasia, 2 with Nijmegen breakage syndrome, 1 with Edwards syndrome, 1 with main classes immunoglobulin deficiency and 1 with C-1 esterase inhibitor and partial C4 component deficiency. Among patients under 3 years old, three with main classes immunoglobulin deficiency had BMI Z-score below -2SD.

## Discussion

Nutritional status is a valuable factor, which has an impact on immunological response [8]. The relationship between nutritional status and the immune system has been a topic of study for much of the twentieth century [9]. With no doubt malnutrition is considered as one of the most common causes of impaired immunity worldwide [10]. As nutritional status has its influence on susceptibility to infections, so may immune system disorders have an influence on nutritional status [11]. Many original papers have proven higher frequency of malnutrition and anthropometric values abnormalities in some of diseases from the PID spectrum [12–14]. Because of heterogeneity of this group, results of each study should be analyzed with inclusion of certain disease characteristics.

Improper nutritional status has been seen in the patients with common variable immunodeficiency (CVID) [12, 13]. Additionally, analyzed anthropometric parameters have indicated higher frequency of malnutrition in these patients’ population, comparing to general population. It has been noticed that patients with CVID had higher susceptibility to infections, persistent, prolongating diarrhea and absorption disorders, which may result in malnutrition of this group, hence causing higher risk of disease complications [12]. A study conducted on Iranian children suffering from humoral immunity disorders, including CVID (52.5% of participants) and X-linked agammaglobulinemia (27.5% of participants) underweight was detected (BMI < 5 percentile) among 21.1% children, with no relevant differences between sexes [13]. Authors noticed that frequency of malnutrition of children with humoral immunity disorders is almost 4 times higher than in the healthy children population, based on data from Center for Disease Control (CDC) of year 2000. Moreover, in this study height and weight relative to age were also improper: 57.5% of patients had too small weight for their age (based on Z-score < − 1) and 63.2% were too short for their age. In a study conducted by Muscaritoli et al., anthropometric measurements allowed to find out that 23% of adults with CVID had BMI < 18,5 and a higher susceptibility to protein-energetic malnutrition [12]. Originally three patients with CVID took part in our study, but we had to exclude two of them due to age over 18 years old. Weight, height and BMI during hospitalization of one patient with CVID remained normal (− 2 < Z-score < + 2; 3 < centile< 97). Four patients with main classes immunoglobulin deficiency (other than CVID) in our study had improper nutritional status according to their BMI (Z-score < − 2SD and/or underweight assessed by centile charts).

1 patient with chronic granulomatous disease took part in our study. During hospitalization his weight, height and BMI remained normal. (− 2 < Z-score < + 2). Cole et al. study compared patients with the disease after hematopoietic stem cell transplantation and without this treatment [15]. Among the assessed parameters were anthropometric ones (height, weight, BMI), which were applied to growth standards developed by WHO. Height and nutritional status disorders were mainly found in the patients without transplantation group. 20% of this group were too short, compared to age (z-score < − 2) and 16.67% had too low BMI relative to age. Weight z-score < −2SD in children without transplantation was found at 22% of patients. Too small group of people under study (*n* = 1) didn’t allow for any clear conclusions about nutritional status of them.

A special group of patients with PID are the ones of chromosomal instability group (ataxia-telangiectasia (AT) and Nijmegen breakage syndrome (NBS). Apart from genetically lowered capability to DNA reparation, these two syndromes are also connected by higher tendency to immune system disorders. Because of the rarity of ataxia-telangiectasia, there are few available sources evaluating physical growth and nutritional status of this disease. In Ehlayel et al. study on 13 patients with AT, 38% were too short, based on the height standard deviation score (HtSDS) < − 2SD. Patients’ BMI was low in 31% of them (BMI SD < − 2) [16]. In our study 7 patients with AT took part. At 28.6% (*n* = 2) BMI < -1 was noticed, at 28.6% (n = 2) BMI < -2SD. A small number of patients affected with disease, as well as high frequency of nutritional status irregularities within the group implies the necessity to conduct further in-depth researches on anthropometric parameters of patients being under constant care of each health centre. Stewart E et al., reported 101 patients specifically on their progressive growth failure and even recommended early proactive consideration of percutaneous endoscopic gastrostomy (PEG) from age 8 years onwards to prevent it [17]. In our study there were no patients with PEG.

Nijmegen breakage syndrome seems to occur worldwide, but with a distinctly higher prevalence among Central European and Eastern European populations [18, 19]. The long-term study of over 70 patients with NBS conducted by Chrzanowska et al. showed retardation in their somatic growth practically since birth [20]. The mean birth anthropometric parameters, including weight, length, head and chest circumferences were significantly lower than in healthy population. Infants of both genders showed a growth deficit until the age of 2 or 3 when some gain of height and weight, but not occipitofrontal circumference was observed. In later stages of childhood and adolescence, differences in the growth pattern between girls and boys became apparent: the growth spurt in boys was poor and was absent in girls. The mean height in over half of the adult girls and boys with NBS was within lower normal ranges. Among 4 patients with Nijmegen breakage syndrome in our study (aged 1,5-11y.), one had BMI < -1SD, second one <−2SD. According to Polish centile charts, nutritional status of these 2 patients was determined as underweight. These irregularities affected girls (*n* = 2), boys were within norm. Both females were also born with birth weight under 2500 g, while boys > 2500 g. In case of that disease, growth retardation, as well as microcephaly are in its symptoms spectrum. Differences between sexes and small numbers of current publications about growth and physical development of children with Nijmegen breakage syndrome suggest the need for further studies of this topic.

Evaluation of physical growth presented in our study on heterogenous group of diseases, which are primary immunodeficiency disorders, shows higher percentage of children with PID improperly developed, compared to the control group and RRTI group. Almost every 5th patient with PID had weight and height < 3 centile, moreover 23% over the age of 3 were underweight. It is worth to mention that improper nutritional status involves not only specific syndromes like Nijmegen breakage syndrome or ataxia-telangiectasia but also predominantly antibody deficiencies. We wanted to pay attention to higher risk of malnutrition in all PID patients, not only in particular conditions, where patients are known to have growth retardation primary or secondary to their disease.

In Adirmaz et al. study 11.2% of children with PID were retarded in their height and physical development and in the study on Egyptian children interrupted physical development and growth were detected among 28% of patients diagnosed with PID [21, 22]. In our study higher percentage of malnourished children in Ig + therapy group can be explained by complexity of diseases’ syndromes, often exceeding over immune system, which may also limit patients’ development in a significant way (Nijmegen breakage syndrome, ataxia-telangiectasia). We believe the topic needs further studies on group of children with immunoglobulin substitution. It’s worth to mention that in many children with PID higher percentage of low birth weight and length is detected, not only in syndromic immune deficiencies. This interesting finding requires further studies as well.

The study was performed as Students Research Circle’s activity. Main limitations of the study were relatively short time-span and lack of data about gestational age of patients.

## Conclusions

Knowledge about primary immunodeficiency disorders have expanded widely during the last few years because of assigning over 200 genes to a certain PID types [2]. Conducted American researches as screening programs of PID occurrence at newborns allow early diagnosis and proper immunotherapy treatment, as well as significantly improving quality of life and stable psychomotor growth of patients [23]. In many countries, including Poland, that kind of screenings aren’t conducted and the subject of PID still remains problematic for many physicians. Propagating knowledge in both medical field and in the society of 10 warning signs which help to initially rule out or to diagnose primary immunodeficiency disorder is a valuable tool for diagnosing these diseases on preliminary, but essential level [24]. Among untreated/undiagnosed patients with PID recurrent infections significantly affect child’s proper growth. Prolonged antibiotic therapy and hospitalizations, associated with infections, can cause nutritional deficiencies, which affect harmonic growth of patients. Therefore it is essential to educate physicians of all specializations about PID, since earlier diagnosis leads to faster proper treatment. Early diagnosis allows implementation of suitable prophylactic procedures and lowers the frequency of infections in that group of patients. It has a significant impact on the quality of life as ambulatory treatment and hospitalizations disturb the execution of their daily duties. Purpose of the medical care is to provide patients with the best care so the disease won’t interfere with their harmonic growth and allow them to live a normal life in the society.

In our study statistically significant differences were detected between RRTI and PID group in the field of birth weight, as well as weight, height and nutritional status during hospitalization. No statistically significant differences were found between RRTI and control group. Heterogenous etiology of RRTI makes implementation of universal procedures for these patients difficult. Each clinical case should be investigated individually, and if possible, the cause of disease should be removed. Presence of physical growth abnormalities in both groups indicates the need to increase awareness (both among medical doctors and society) of factors which may influence the parameters mentioned above (height, weight, BMI), as well as immune system irregularities among children.

## Methods

The study was conducted utilizing retrospective analysis. Primary research material were medical histories of patients hospitalized in Clinical Immunology and Pediatrics Ward, J. Gromkowski Provincial Clinical Hospital in Wrocław from October 2014 up to March 2015.

The study group numbered 195 people (*n* = 96 female, *n* = 99 male), whose age ranged from 4 weeks old up to 18 years old. The group was divided into 3, which is shown at Table S1 [supplementary file 1]:

- □ PID (*n* = 56); largest group were patients with predominantly antibody deficiencies (*n* = 41). Most of the patients were diagnosed with IgG deficiency (*n* = 15; 26.8%) and IgG subclass deficiency (*n* = 12; 21.4%). There was one patient with X-linked agammaglobulinemia (XLA). No patients with combined immunodeficiency were hospitalized in our clinic during data collection. Furthermore, PID was divided in terms of applied treatment - with immunoglobulin replacement therapy (*n* = 30, 53.6%) and without (*n* = 26, 46.4%) (Table S2). Despite patients over the age of 18 were hospitalized in clinic during data collection, we decided to exclude them from the study group (*n* = 5).
- □ Among recurrent respiratory tract infections group (RRTI; *n* = 69), no abnormalities in terms of immunological tests were found, but had a history of mild recurrent upper respiratory tract infections at least 8 times in 12 months and required immunological diagnostics. These patients were admitted to our clinic at the same time as PID group to conduct immunological tests. Patients with chronic conditions (eg. cystic fibrosis, congenital heart defects, diagnosed gastroesophageal reflux disease), which may have an impact on recurrent respiratory tract infections, were excluded from the study.
- □ Control group (*n* = 70); children with occasional infections, their cause of hospitalization were: random poisoning (31%), acute pharyngitis (19%), acute gastroenteritis (10%) and other causes (40%). No history of recurrent infections (at least 8 times in 12 months) was detected.

Patients with PID treated with immunoglobulin (PID Ig + therapy) were admitted to the clinic every month or every 3 months. For adequate groups comparison, data were collected at the same time as PID patients were hospitalized.

Assessed parameters

- □ Birth length and weight
- □ Height and weight during admission to the hospital
- □ Body mass index (BMI)

Due to insufficient amount of patients’ data about gestational age at birth, we have not assessed birth length and weight with use of centiles. We have acknowledged a cut-off point at 2500 g. We decided to exclude patients with Nijmegen breakage syndrome from birth length and weight analysis. Analyzing height and weight during the admission, each parameter was assigned to corresponding centile intervals: < 3, 3–10, 10–25, 25–50, 50–75, 75–90, 90–97, > 97 centile. These values were assessed using development standards analyzed and published for the Polish children population [25] as well as WHO standards [26]. Polish development standards allowed to evaluate the development of the whole study group, based on the centile charts. Values below 3rd centile were interpreted as abnormal [27]. BMI was calculated as weight in kilograms divided by height in meters squared: BMI = weight (kg) ÷ height (m)^2^. The BMI was defined as: underweight, normal weight, overweight and obese, based on the latest development standards of BMI published in Poland for age 3–18 years old [25]. According to the authors overweight and obesity were defined using the BMI cut-offs given in the international definition of obesity for children and adolescents [28], whereas underweight, on the basis of the BMI cut-offs proposed by Cole TJ et al. [29, 30]. Furthermore, we assessed BMI’s z-score, according to WHO recommendations, as incorrect when situated <− 2SD (underweight), <−3SD (severely underweight), >2SD (overweight), >3SD (obesity). Z-score was assessed for such values as: height and BMI relevant to age on the basis of WHO standards [26] for all the patients. Both weight centiles and z-score according to WHO standards were available only for ages 0–10 years old, therefore evaluation of these parameters was excluded with WHO standards.

Statistical analysis of data was conducted using the spreadsheet of the Microsoft Office Excel 2010 (Microsoft Corp. Washington, WA, USA) and Statistica 12 – for categorical data – Pearson’s chi-squared; for continuous data – Student’s t-test and Mann-Whitney U test.

The study had a consent of a bioethical commission.

## Supplementary information

**Additional file 1: Supplementary file 1.** Tables and figures.

**Additional file 2: Supplementary file 2.** Data generated and analysed during the study.

## Data Availability

All data generated or analysed during this study are included in this published article [and its supplementary information files].

## References

[1] Ballow M, Notarangelo L, Grimbacher B, Cunningham-Rundles C, Stein M, Helbert M (2009). Immunodeficiencies. Clin Exp Immunol.

[2] Picard C, Bobby Gaspar H, Al-Herz W, Bousfiha A, Casanova JL, Chatila T (2018). International Union of Immunological Societies: 2017 primary immunodeficiency diseases committee report on inborn errors of immunity. J Clin Immunol.

[3] Kilic SS (2004). Recurrent respiratory tract infection. Recent advances in pediatrics.

[4] Raniszewska A, Górska E, Kotuła I, Stelmaszczyk-Emmel A, Popko K, Ciepiela O (2015). Recurrent respiratory tract infections in children - analysis of immunological examinations. Cent Eur J Immunol.

[5] Jenesak M, Ciljakova M, Rennerova Z, et al. Recurrent Respiratory Infections in Children – Definition, Diagnostic Approach, Treatment and Prevention. In: Martin-Loeches I, editor. Bronchitis. InTech; 2011. p. 119–148. https://www.intechopen.com/books/bronchitis/recurrent-respiratory-infections-in-children-definition-diagnostic-approach-treatment-andprevention, 10.5772/19422.

[6] Lankisch P, Schiffner J, Ghosh S (2015). The Duesseldorf warning signs for primary immunodeficiency: is it time to change the rules?. J Clin Immunol.

[7] Glocker E, Ehl S, Grimbacher B (2007). Common variable immunodeficiency in children. Curr Opin Pediatr.

[8] Corman LC (1985). The relationship between nutrition, infection, and immunity. Med Clin North Am.

[9] Keusch GT (2003). The history of nutrition: malnutrition, infection and immunity. Symposium: nutrition and infection, prologue and Progress since 1968. J Nutr.

[10] Chandra RK (1997). Nutrition and the immune system: an introduction. Am J Clin Nutr.

[11] Scrimshaw NS, San Giovanni JP (1997). Synergism of nutrition, infection, and immunity: an overview. Am J Clin Nutr.

[12] Muscaritoli M, Fanfarillo F, Luzi G, Sirianni MC, Iebba F, Laviano A (2001). Impaired nutritional status in common variable immunodeficiency patients correlates with reduced levels of serum IgA and of circulating CD4+ T lymphocytes. Eur J Clin Investig.

[13] Kouhkan A, Pourpak Z, Moin M, Dorosty AR, Safaralizadeh R, Teimorian S (2004). A study of malnutrition in Iranian patients with primary antibody deficiency. Iran J Allergy Asthma Immunol.

[14] Luzi G, Zullo A, Iebba F, Rinaldi V, Sanchez Mete L, Muscaritoli M (2003). Duodenal pathology and clinical immunological implications in common variable immunodeficiency patients. Am J Gastroenterol.

[15] Cole T, Pearce MS, Cant AJ, Cale CM, Goldblatt D, Gennery AR (2013). Clinical outcome in children with chronic granulomatous disease managed conservatively or with hematopoietic stem cell transplantation. J Allergy Clin Immunol.

[16] Ehlayel M, Soliman A, De Sanctis V (2014). Linear growth and endocrine function in children with ataxia telangiectasia. Indian J Endocrinol Metab.

[17] Stewart E, Prayle AP, Tooke A (2016). Growth and nutrition in children with ataxia telangiectasia. Arch Dis Child.

[18] Chrzanowska KH, Kleijer WJ, Krajewska-Walasek M, Białecka M, Gutkowska A, Goryluk-Kozakiewicz B (1995). Eleven polish patients with microcephaly, immunodeficiency, and chromosomal instability: the Nijmegen breakage syndrome. Am J Med Genet.

[19] Kondratenko I, Paschenko O, Polyakov A, Bologov A (2007). Nijmegen breakage syndrome. Adv Exp Med Biol.

[20] Chrzanowska K, Kalina M, Rysiewski H, Gajdulewicz M, Szarras-Czapnik M, Gajtko-Metera M (2010). Growth pattern in patients with Nijmegen breakage syndrome: evidence from a longitudinal study. Horm Res Paediatr.

[21] Aldirmaz S, Yucel E, Kiykim A, Çokuğraş H, Akçakaya N, Camcıoğlu Y (2014). Profile of the patients who present to immunology outpatient clinics because of frequent infections. Turk Pediatri Ars.

[22] Reda SM, Afifi HM, Amine MM (2009). Primary immunodeficiency diseases in Egyptian children: a single-center study. J Clin Immunol.

[23] Ballow M (2014). Historical perspectives in the diagnosis and treatment of primary immune deficiencies. Clin Rev Allerg Immunol.

[24] Lewandowicz-Uszyńska A, Pasternak G, Kuraszewicz A, Prościak M, Gul K, Lewicka P, Pirogowicz I. Pierwotne niedobory odporności u dzieci - obraz kliniczny [Primary immunodeficiency - clinical characteristics]. In:Iwona Pirogowicz, Barbara Iwańczak, Aleksandra Lewandowicz-Uszyńska. Dziecko - jego zdrowie i jego środowisko: objawy alarmowe w pediatrii z perspektywy gastroenterologa, ginekologa i immunologa kliniczego [A child - its health and enviroment: warning symptoms in pediatrics - gastroenterologist's, gynecologist's and immunologist's perspective]. 1st ed. Wrocław: Wrocławskie Wydawnictwo Naukowe Atla 2; 2017. p.13–21.

[25] Kułaga Z, Różdżyńska-Świątkowska A, Grajda A, Gurzkowska B, Wojtyło M, Góźdź M, Świąder-Leśniak A, Litwin M (2015). Percentile charts for growth and nutritional status assessment in polish children and adolescents from birth to 18 year of age. Stand Med Ped.

[26] WHO Multicentre Growth Reference Study WHO Child Growth Standards (2006). Length/height-for-age, weight-for-age, weight-for-length, weight-for-height and body mass index-for-age: Methods and development.

[27] A health professional’s guide for using the new WHO growth charts. Paediatr Child Health. 2010;15(2):84–98. 10.1093/pch/15.2.84 PMC2865941 21286296

[28] Cole TJ, Bellizzi MC, Flegal KM, Dietz WH (2000). Establishing a standard definition for child overweight and obesity worldwide: international survey. BMJ.

[29] Cole TJ, Flegal KM, Nicholls D, Jackson AA (2007). Body mass index cut offs to define thinness in children and adolescents: international survey. BMJ..

[30] Grajda A, Kułaga Z, Gurzkowska B, Napieralska E, Litwin M (2011). Regional differences in the prevalence of overweight, obesity and underweight among polish children and adolescents. Med Wieku Rozw.

