# Supplementary material for: Assessment of weight and height of patients with primary immunodeficiency disorders and group of children with recurrent respiratory tract infections
Source: BMC Immunol. 2020 Jul 16;21:42. doi: 10.1186/s12865-020-00372-x (PMC7364511; doi:10.1186/s12865-020-00372-x)

Supplementary Material

Assessment of weight and height of patients with primary immunodeficiency disorders and group of children with recurrent respiratory tract infections.

Karolina Pieniawska-Śmiech, Kamil Bar, Mateusz Babicki, Karol Śmiech, Aleksandra Lewandowicz-Uszyńska

Tables

Table S1. Study groups characteristics.

| **Group** | **Patients' age** | **Number of patients** | **Female patients** | **Male patients** |
| --- | --- | --- | --- | --- |
| PID | < 1 yo = 4  1-3 yo = 14  3-6 yo = 12  6-12 yo = 17  12-18 yo = 9  (median = 6 yo) | 56 | 29 | 27 |
| RRTI | < 1 yo = 1  1-3 yo = 23  3-6 yo = 26  6-12 yo = 13  12-18 yo = 6  (median = 4 yo) | 69 | 33 | 36 |
| Control group | < 1 yo = 15  1-3 yo = 20  3-6 yo = 11  6-12 yo = 16  12-18 yo = 8  (median = 30 mo) | 70 | 34 | 36 |
| Total | 4 weeks - 18 years | 195 | 96 | 99 |

Table S2. Clinical characteristics of patients with PID.

| Primary immunodeficiency (n=56) | Number of patients |
| --- | --- |
| Predominantly antibody deficiencies | 41 |
| Ataxia-telangiectasia | 7 |
| Nijmegen breakage syndrome | 4 |
| DiGeorge syndrome | 1 |
| Kabuki syndrome | 1 |
| Chronic granulomatous disease | 1 |
| C1 esterase inhibitor and partial C4 component deficiency | 1 |

Figure-caption list

Figure S1. Birth weight and length analysis results as mean values ± standard deviations, PID divided into group with immunoglobulin substitution (PID Ig+therapy) and without (PID Ig-therapy). * - p<0.05; ** - p<0.01; *** - p<0.001.


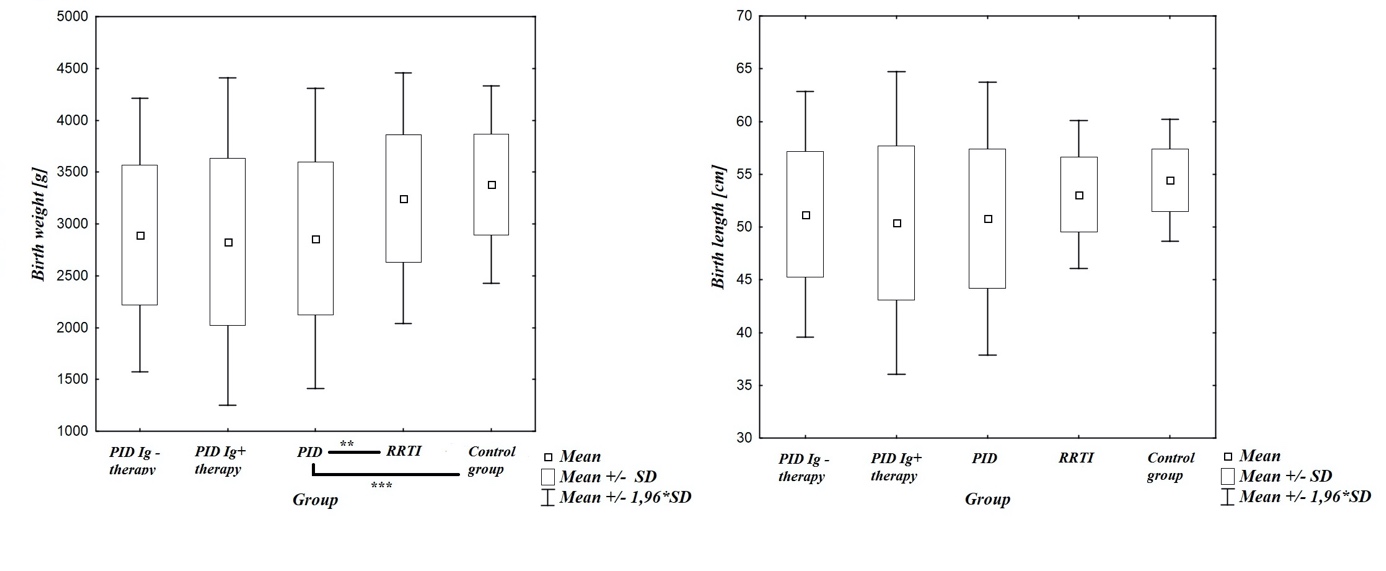


Figure S2. Centiles percent distribution of certain patients groups actual weights, according to Polish standards.


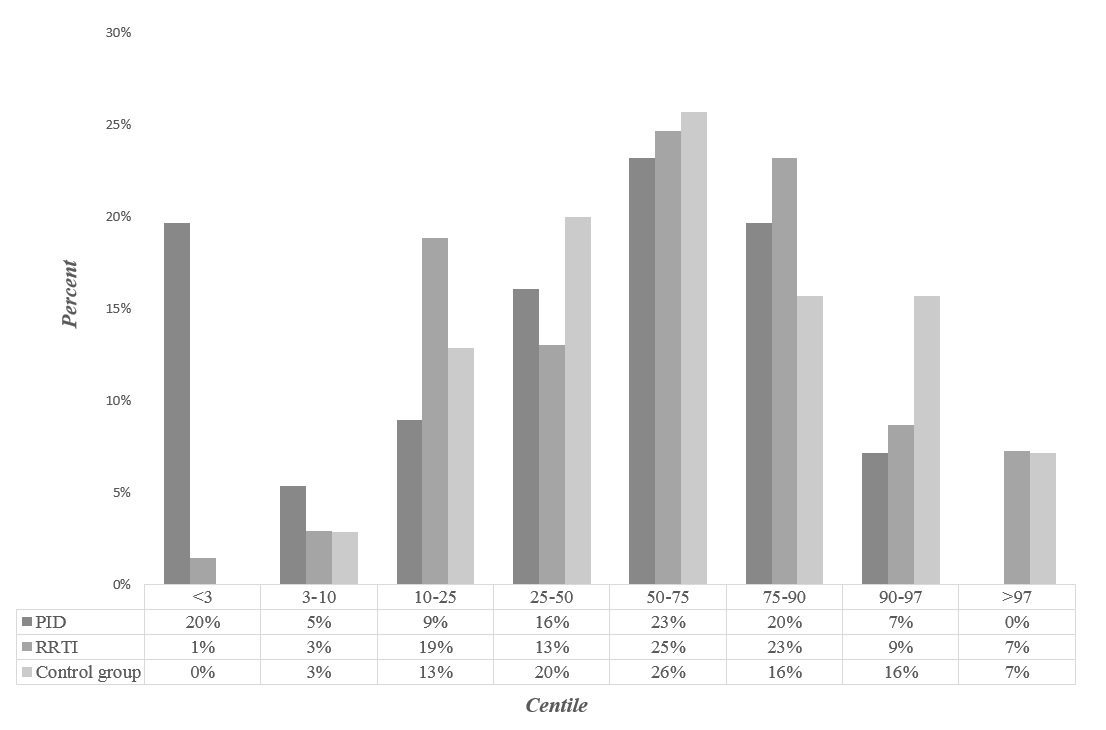


Figure S3. Centiles percent distribution of certain patients groups height, according to Polish standards.


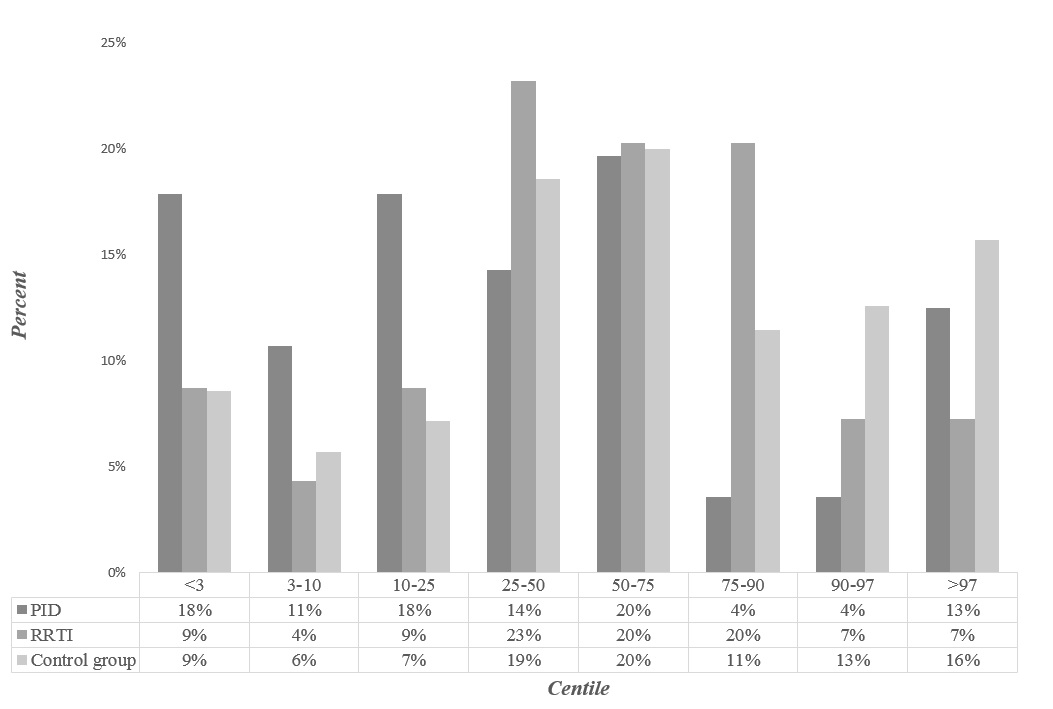


Figure S4. Z-score values of height of the PID, RRTI and control group patients.


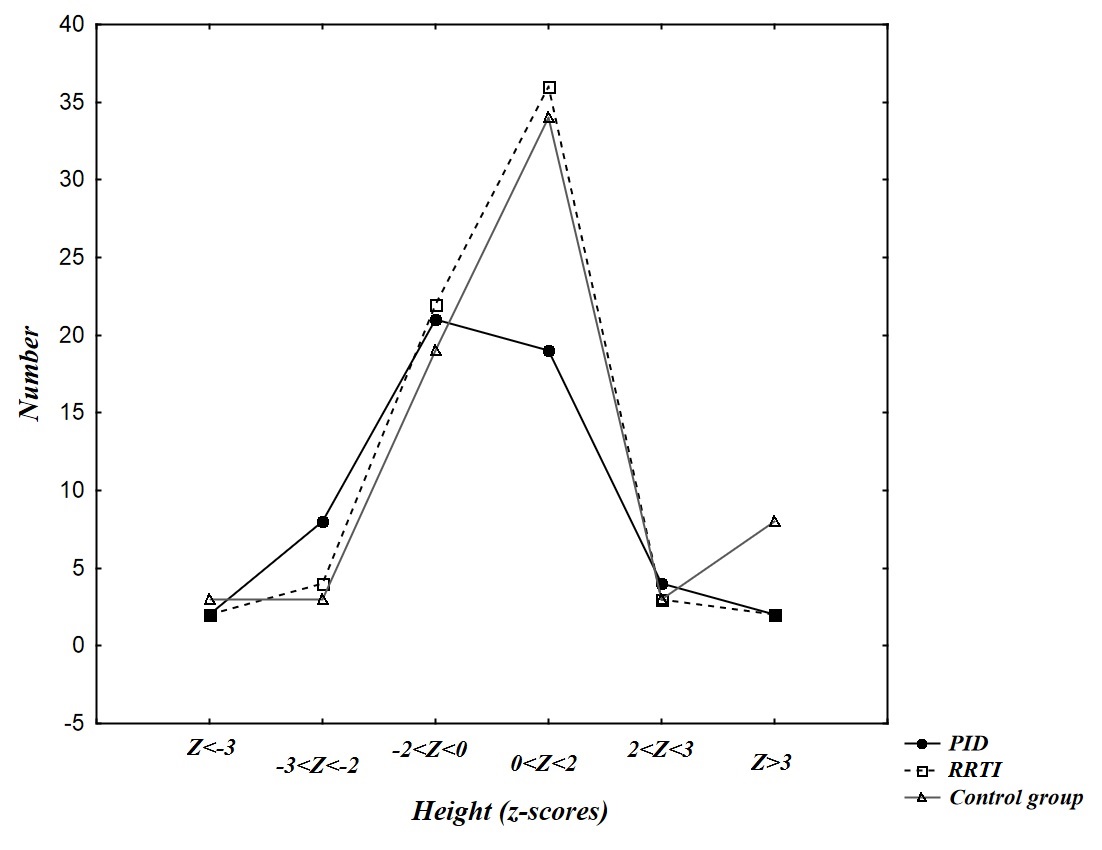


Figure S5. Nutritional status of participants aged 3-18 years old divided into groups.


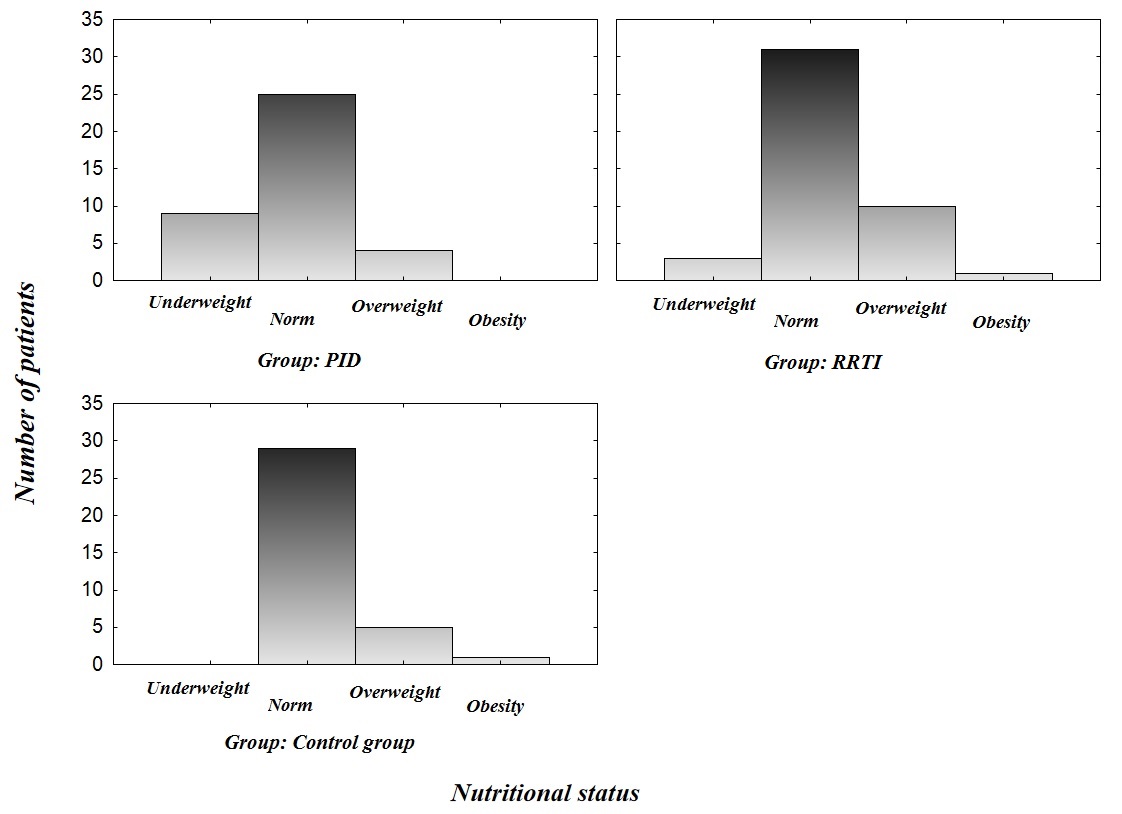


Figure S6. BMIs Z-score of the PID, RRTI and control group.


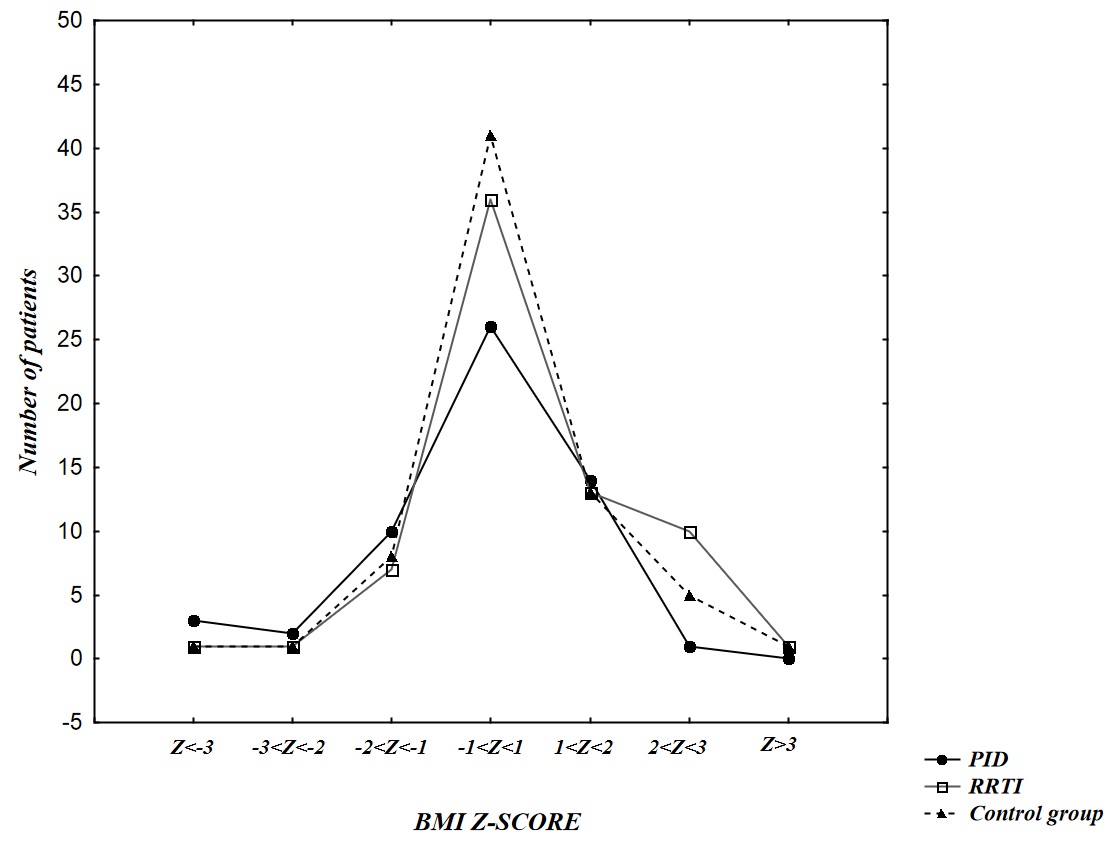


Figure S7. Nutritional status of PID patients group divided into Ig+therapy and Ig-therapy groups.


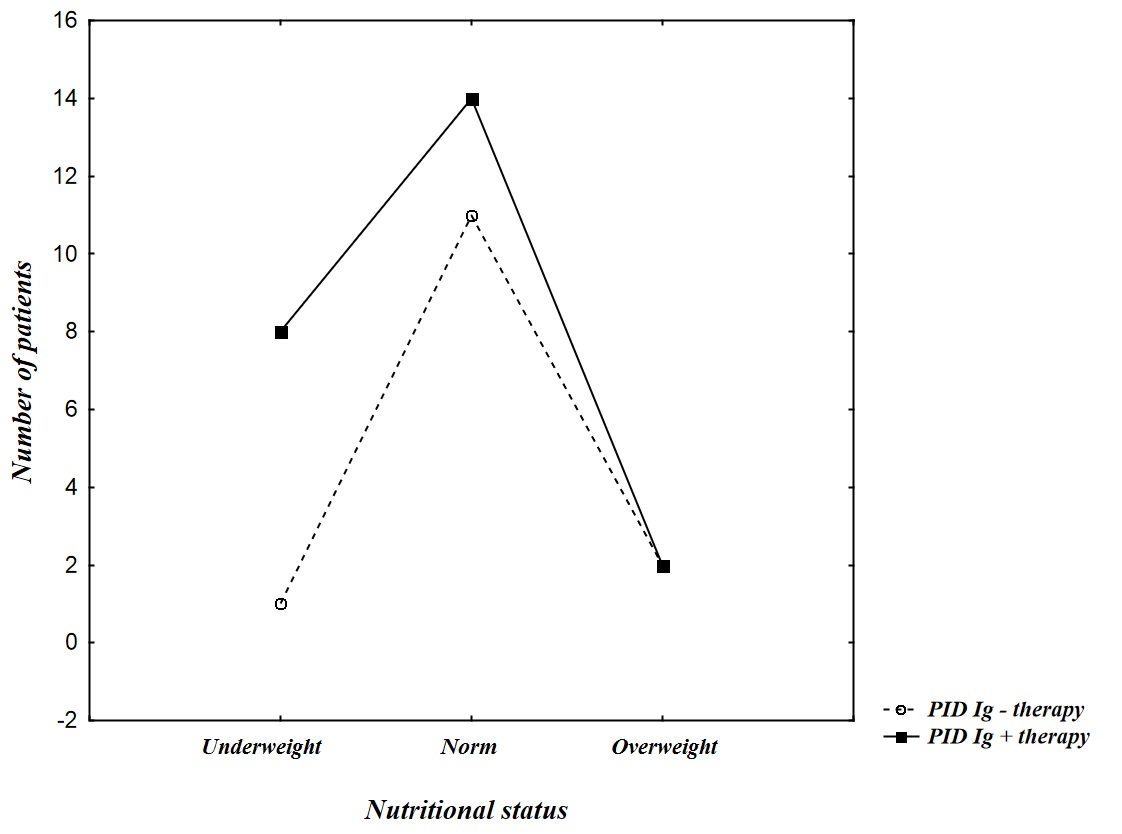

Supplement: Supplementary file 1 — Additional file 1: Supplementary file 1. Tables and figures. [file 12865_2020_372_MOESM1_ESM.docx]
